# A Case of Unresolved and Worsening Retroperitoneal Abscess

**DOI:** 10.1155/2018/6740734

**Published:** 2018-01-16

**Authors:** Raghav Bansal, Mohamed Barakat, Soohwan Chun, Sonam Rosberger, Joel Baum, Melik Tiba

**Affiliations:** ^1^Division of Gastroenterology and Hepatology, Icahn School of Medicine at Mount Sinai, Elmhurst Hospital Center, Elmhurst, NY, USA; ^2^Department of Internal Medicine, Icahn School of Medicine at Mount Sinai, Queens Hospital Center, Jamaica, NY, USA; ^3^Department of Radiology, Icahn School of Medicine at Mount Sinai, Elmhurst Hospital Center, Elmhurst, NY, USA

## Abstract

Retroperitoneal abscess is a rare condition which is difficult to diagnose and treat because of its insidious onset. Herein, we present a case of retroperitoneal abscess secondary to a perforation that occurred during an ERCP. A 54-year-old female patient was admitted to an outside hospital with gallstone pancreatitis and underwent ERCP with sphincterotomy followed by laparoscopic cholecystectomy. An abdominal CT scan was performed at the outside hospital 10 days later for worsening abdominal pain which showed multiple loculated pockets in the right upper and lower quadrant. Her condition improved after IV antibiotics and percutaneous drainage. Her symptoms recurred a month later and she presented to our hospital. Repeat abdominal CT scan at our hospital revealed recurrence of her abscesses. Multiple drains were placed and the abscess cavity was washed out without much improvement. EGD revealed a small mucosal defect in the distal portion of the duodenal bulb which was closed successfully using an over-the-scope clip. Repeat CT scan after 8 weeks from the endoscopic closure showed near complete resolution of the abscess. ERCP-associated perforation is a rare complication and can be challenging to diagnose and treat; prompt recognition is mandatory for favorable prognosis. Our patient was managed successfully via nonsurgical approach.

## 1. Introduction

Retroperitoneal abscess is a rare condition which is difficult to diagnose and treat because of its insidious onset and nonspecific clinical manifestations. Retroperitoneal abscess may result from a variety of causes, such as pyelonephritis, pancreatitis, retroperitoneal appendicitis, diverticulitis, peptic ulcer disease, perforated cancer, inflammatory bowel disease, spinal infection, trauma, and postinstrumentation. Herein, we report a unique case of retroperitoneal abscess in a 54-year-old female and its management.

## 2. Case Report

A 54-year-old female patient was admitted to an outside hospital with gallstone pancreatitis and underwent endoscopic retrograde cholangiopancreatography (ERCP) with sphincterotomy followed by laparoscopic cholecystectomy. A hepatobiliary scan (HIDA) was performed on postoperative day 5 for worsening abdominal pain and failed to reveal any evidence of bile leak or obstruction. Abdominal computed tomography (CT) scan with i.v. contrast revealed normally enhancing pancreas along with multiple loculated pockets in the right upper and lower quadrant. The patient was started on i.v. antibiotics and a total of 800 ml of fluid was aspirated via percutaneous drainage. The patient's condition eventually improved and she was discharged home with a Jackson Pratt (JP) catheter in place. Her symptoms recurred after 1 month and she presented to our hospital.

**Figure 1:**
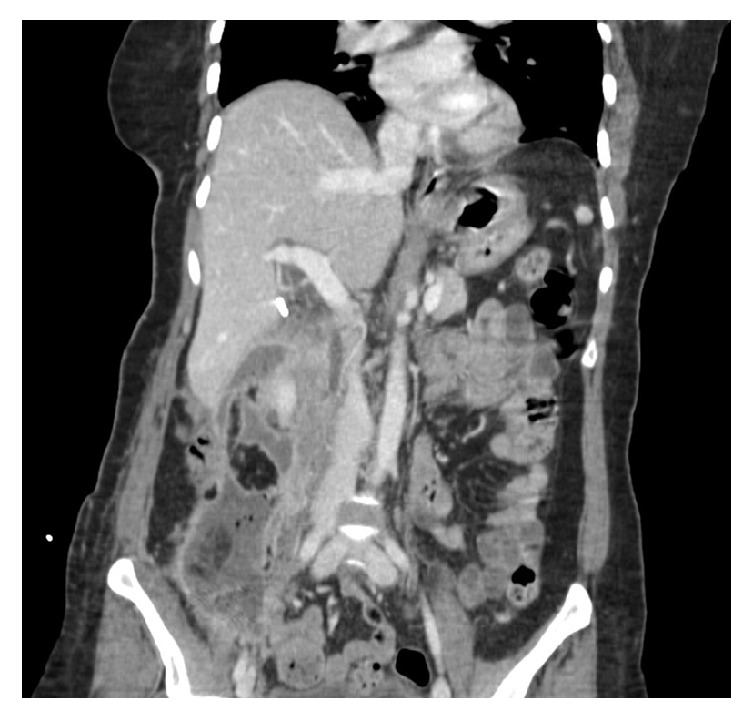
Coronal abdominal CT scan image showing retroperitoneal abscess.

The initial computed tomography (CT) abdomen at our hospital revealed a loculated air and fluid filled, rim enhancing collection in the right retroperitoneum extending to the pelvis measuring approximately 20.8 × 6.7 × 5.3 cm with a JP drain (Figure 1). Another loculated 2.5 × 1.9 cm collection was seen in the region of the gallbladder fossa and right extraperitoneal perivesical component measuring 8.0 × 2.4 cm. The drain was suspected to be clogged and was subsequently replaced with a new JP catheter. The pancreas appeared normal on imaging.

Culture from the abscess grew multiple bacteria:* Haemophilus parainfluenzae*,* Streptococcus anginosus*,* Pseudomonas aeruginosa*, and* Eikenella corrodens*. The presence of multiple gut-derived bacteria was concerning for possible gastrointestinal tract injury during either ERCP or laparoscopic cholecystectomy. There was no obvious evidence of extraluminal air or oral contrast extravasation on imaging. Given patient's overall stable condition, the abscess was managed conservatively with broad spectrum intravenous antibiotics (piperacillin and tazobactam) and percutaneous drainage.

**Figure 2:**
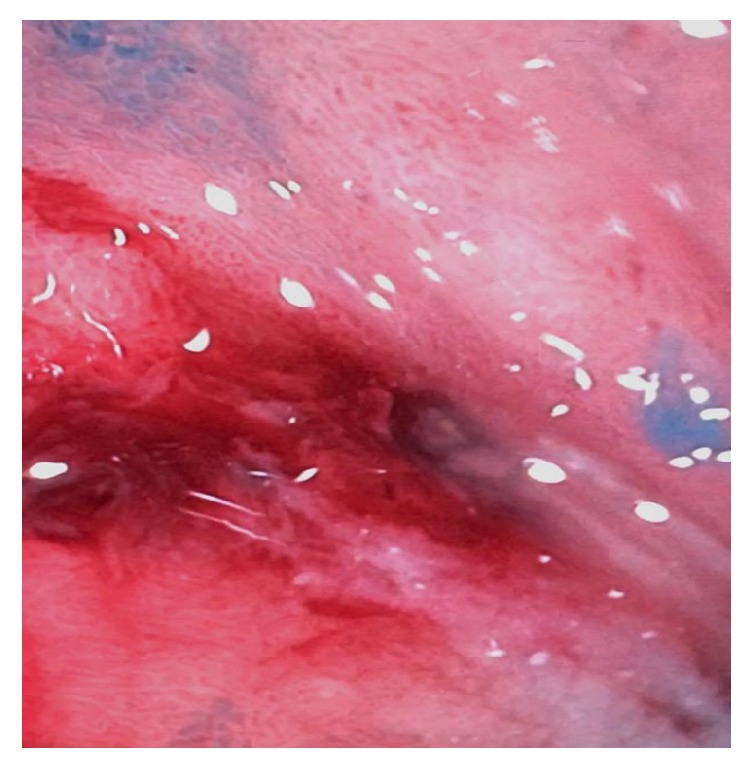
Fistulous opening in the duodenum.

**Figure 3:**
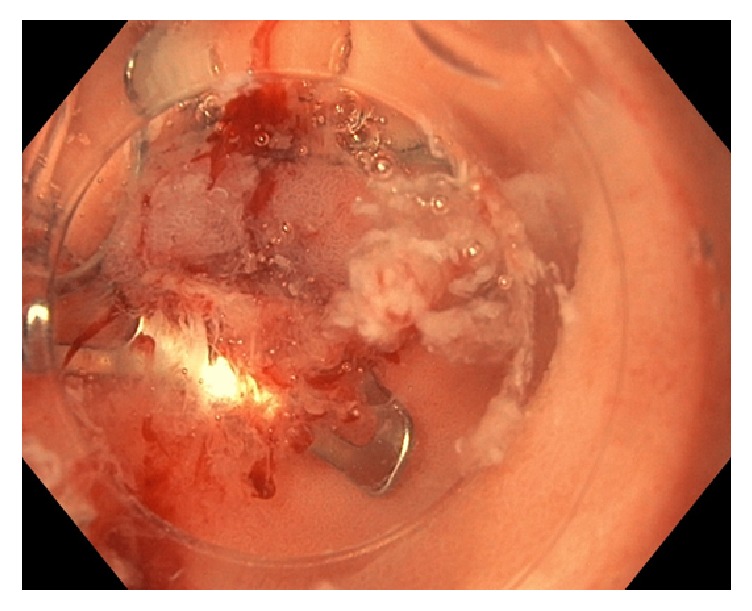
Endoscopic closure of luminal perforation of the duodenum with OVESCO over-the-scope clip.

At 2 weeks, repeat CT scan of the abdomen and pelvis showed no significant change in size of the abscess with persistence of multiple loculations. The patient subsequently underwent surgical drainage and wash-out. However, another repeat CT scan of the abdomen and pelvis two weeks later showed recurrence of the retroperitoneal abscess. Amylase and lipase levels were tested in the fluid drained from the abscess and were found to be elevated to 1335 units/L and 1655 units/L, respectively. Both magnetic resonance cholangiopancreatography (MRCP) and HIDA scan did not show any evidence of biliopancreatic ductal disruption and was confirmed with a normal cholangiogram during repeat ERCP. At this point, a nonhealing gastrointestinal tract perforation was strongly suspected. Finally, an upper endoscopy was performed after 12 weeks of initial admission revealed a small mucosal defect in medio-posterior wall of distal portion of the duodenal bulb that was draining pus intermittently, suspicious for the site of perforation (Figure 2). Methylene blue was injected through one of the JP catheters and was noted to be intravasating into the lumen via the defect, confirming communication with the retroperitoneal abscess. An OVESCO over-the-scope clip (12/6T) was used to close the defect and a second injection of methylene blue via the catheter did not show further luminal drainage (Figure 3).

**Figure 4:**
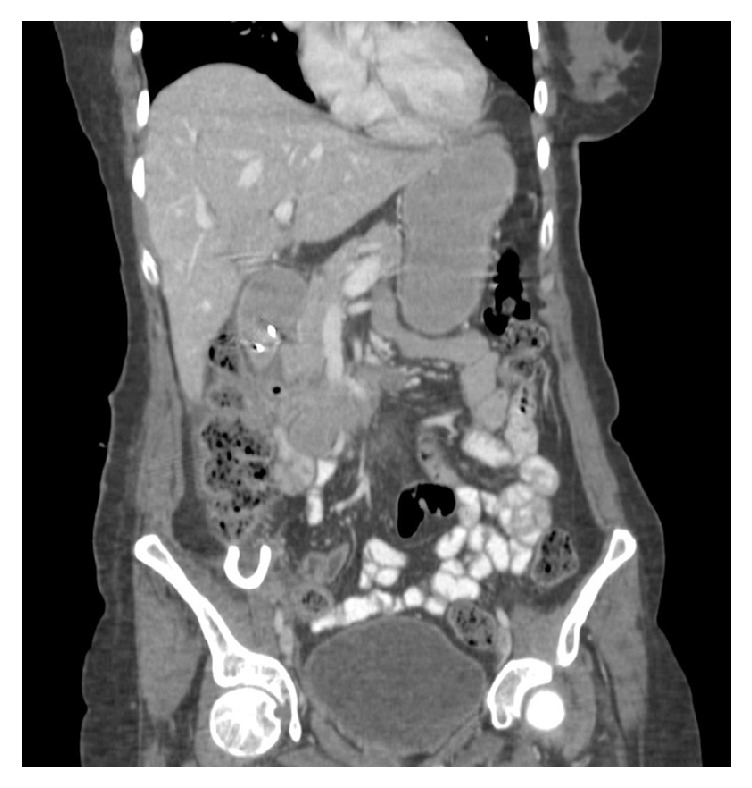
Coronal abdominal CT scan images after the closure of the fistula.

The patient's condition improved gradually with significant reduction in size of the abscess at 6 weeks from the clip placement. Repeat CT scan after 8 weeks from the endoscopic closure showed near complete resolution of the abscess (Figure 4).

## 3. Discussion

ERCP-associated perforation is a rare complication that occurs in less than 1% of cases [1]. It is associated with significant morbidity and mortality; prompt recognition is central to the management as earlier intervention is associated with improved survival [2]. Our case reflects significantly delayed recognition of a probable post-ERCP perforation and highlights the importance of having high index of suspicion despite negative radiographic studies.

ERCP-associated perforation has been traditionally classified into four types according to Stapfer classification, based on its location and mechanism of injury [3, 4]. Type 1 refers to the perforation occurring in lateral (more often) or medial wall of duodenum caused by the side-viewing duodenoscope. Type 2 perforation occurs in periampullary region, most frequently due to sphincterotomy or precut papillotomy. Type 3 refers to disruption of the biliary or pancreatic duct due to instrumentation such as with a guidewire or during stone extractions. Type 4 is a retroperitoneal microperforation, occurring in the setting of excessive insufflation while manipulating the sphincter.

Emergent surgical intervention is required for patients who have signs of peritonitis and intraperitoneal air, suggestive of an intraperitoneal perforation. Patients with retroperitoneal perforation will lack peritoneal signs, present less acutely, and may allow medical management as an initial approach. Perforations associated with retroperitoneal collections generally carry a worse prognosis [4, 5]. Type 1 perforations often tends to result in a large defect with intraperitoneal leakage and has been traditionally managed surgically [6]. Type 2 perforations are the most common, small, and retroperitoneal and can be initially managed medically with intravenous antibiotics and nasogastric tube decompression with or without percutaneous drainage [6, 7]. Type 3 perforations are often recognized during the procedure via fluoroscopy and managed with the placement of biliary or pancreatic plastic stents. Type 4 perforations are often asymptomatic, found incidentally, and typically do not require invasive intervention.

Various treatment algorithms have been suggested based on the type of perforation. Kumbhari et al. [7] proposed surgery as the best initial approach for Type 1 perforation, whereas for Type 2 lesion, surgical intervention was suggested only for refractory patients after a trial of maximal medical therapy. Endoscopic repair has been reported successful and can be attempted in stable patients with both Type 1 and Type 2 perforation. Endoscopic clip placement may suffice for a small Type 1 lesion; larger lesions have been successfully closed with adjunctive use of an endoloop, fibrin glue, and over-the-scope stitch system and more recently with use of an over-the-scope clip [2, 7–11]. Endoscopic band ligation has also been used successfully for closure and may be useful for a defect in a difficult location, given the ease of its application [8].

For Type 2 perforation, biliary plastic stent placement has been performed for biliary diversion and was suggested for a large defect at index endoscopy [12]. More recently, fully covered self-expanding metal stent has been used successfully at both index and repeat (within a few days) endoscopy [13, 14]. Larger final diameter and greater opposing force may confer improved seal at the perforation site. Repeat endoscopy for removal of the stent was successful without increased risk of stent migration in most cases, when performed within 1 month. [14]. Though the scope clips have been used for Type 2 perforation, their application can be challenging using side-viewing endoscope [15]. OTSC similarly may not be ideal for its application, given the acute angulation and potential risk of ampullary orifice closure [15]. Types 3 and 4 lesions were recommended to be managed nonsurgically, in agreement with other previous authors.

Multidetector computed tomography (MDCT) scan is considered highly sensitive in detecting gastrointestinal perforation, whether intraperitoneal or extraperitoneal [16, 17]. Extraluminal air is the most common and consistent finding of gastrointestinal perforation; MDCT is able to detect tiny bubbles of air that plain film may fail to show in as high as 50% of the cases. Focal bowel wall defect with an abrupt adjacent wall thickening and surrounding stranding may be seen. Direct visualization of oral contrast extravasation is an infrequent finding. Based on the focus of abnormality and distribution of the extraluminal air, MDCT can often localize the site of retroperitoneal perforation. For example, right para-renal collection of retroperitoneal air indicates perforation in duodenum or ascending colon, whereas left para-renal collection indicates defect in descending or sigmoid colon: bilateral distribution can be seen in rectal perforation. Our patient had right-sided para-renal distribution of the retroperitoneal abscess but had no evidence of extraluminal air or focal bowel wall abnormalities on imaging, thus making the diagnosis challenging.

Our patient had perforation in distal portion of the duodenal bulb, far from the native ampulla: thus, a Type 1 perforation. The diagnosis was delayed in our case due to lack of typical findings of perforation on the CT scans and contained nature of the perforation.

Elevated pleural amylase level is frequently used to guide the diagnosis in the case of esophageal perforation [18]; however, the relationship between elevated retroperitoneal amylase and/or lipase level and duodenal perforation is unclear. Croce et al. [19] in a case series reported an increase in amylase concentration in bile in a patient with a duodenal perforation resulting from cholecystectomy. Significantly elevated levels of amylase and lipase in our patient likely reflected leakage of the enzymes from the duodenal lumen into the retroperitoneal space via the area of perforation similar to the previously reported case. It is important to exclude other etiologies that can give rise to elevated amylase and lipase in the retroperitoneal collection, such as pancreatic duct disruption or fluid collection in patients with severe pancreatitis. Some bacteria and fungi produce amylase and/or lipase and can cause elevation of the enzymes in an infected collection making it less specific, in the setting of an abscess. It is unknown whether degree of the enzyme elevation is relevant in differentiating the underlying etiologies.

Many of the ERCP-associated perforations are found during the index procedure. The vast majority of the initially unrecognized perforations are further diagnosed via imaging studies. Delayed diagnosis is rare but may occur in patients with small and contained retroperitoneal perforation. Endoscopy is not only seldom required but also is not a routinely recommended diagnostic tool for perforation, given concern for worsening of clinical condition and delay in definitive management. Upon upper endoscopy, an obvious luminal defect may be found; however, small defects may also be unnoticed and thus require a careful examination.

Endoscopic repair with fibrin glue, clips, and over-the-scope clip or stitch with or without biliopancreatic stents has been described for both Type 1 and Type 2 perforations [8–11]. In majority of the cases, the repair was performed during the same index procedure. Jin et al. suggested endoscopic repair to be considered if the diagnosis was made within 6 hours [2]. Artifon et al. showed in their randomized control trial of 23 patients that endoscopic repair within 12 hours was able to close the defect as safely and effectively as a surgical approach [20]. The study did not characterize the perforations based on Stapfer classification; however, it was mentioned that approximately 40% were intraperitoneal. Majority of defects were less than 10 mm; however, up to 9% were larger than 20 mm. Despite the literature, some endoscopists might not feel comfortable pursuing an endoscopic repair for ERCP-associated perforations.

**Figure 5:**
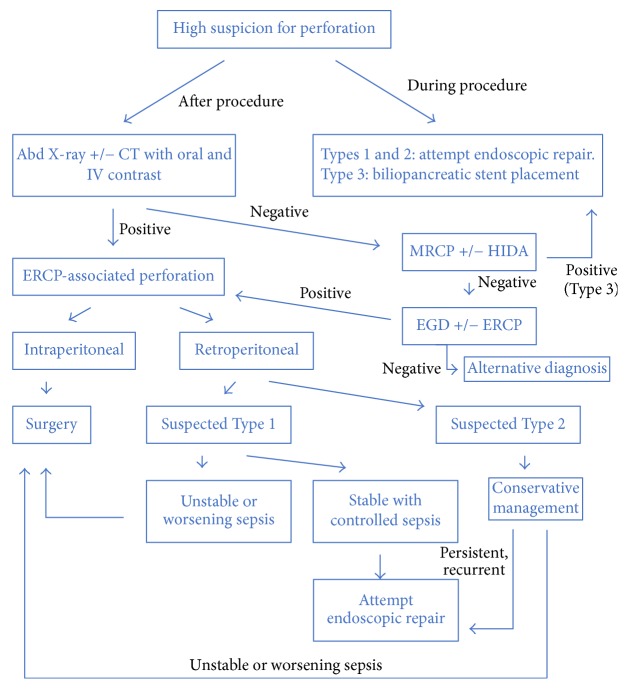
The proposed treatment algorithm for ERCP-associated perforation.

High index of suspicion is required for a prompt diagnosis of ERCP-associated perforations. We propose an algorithm for the work-up and management of ERCP-associated perforations (Figure 5). Overall clinical stability and unremarkable work-up influenced our decision to perform diagnostic and therapeutic endoscopy. Endoscopic repair should be considered even if the diagnosis is significantly delayed—in our case, 10 weeks from the index ERCP.

## References

[1] Andriulli A., Loperfido S., Napolitano G. (2007). Incidence rates of post-ERCP complications: a systematic survey of prospective studies. *American Journal of Gastroenterology*.

[2] Jin Y. J., Jeong S., Kim J. H. (2013). *Clinical Course and Proposed Treatment Strategy for ERCP-Related Duodenal Perforation: a Multicenter Analysis Endoscopy*.

[3] Howard T. J., Tzujen T., Lehman G. A. (1999). Classification and management of perforations complicating endoscopic sphincterotomy. *Surgery*.

[4] Stapfer M., Selby R. R., Stain S. C. (2000). Management of duodenal perforation after endoscopic retrograde cholangiopancreatography and sphincterotomy. *Annals of Surgery*.

[5] Sarr M. G., Fishman E. K., Milligan F. D., Siegelman S. S., Cameron J. L. (1986). Pancreatitis or duodenal perforation after peri-Vaterian therapeutic endoscopic procedures: Diagnosis, differentiation, and management. *Surgery*.

[6] Cirocchi R., Kelly M. D., Griffiths E. A. (2017). A systematic review of the management and outcome of ERCP related duodenal perforations using a standardized classification system. *The Surgeon*.

[7] Kumbhari V., Sinha A., Reddy A. (2016). Algorithm for the management of ERCP-related perforations. *Gastrointestinal Endoscopy*.

[8] Park S. M. (2016). Recent advanced endoscopic management of endoscopic retrograde cholangiopancreatography related duodenal perforations. *Clinical Endoscopy*.

[9] Baron T. H., Gostout C. J., Herman L. (2000). Hemoclip repair of a sphincterotomy-induced duodenal perforation. *Gastrointestinal Endoscopy*.

[10] Mutignani M., Iacopini F., Dokas S. (2006). Successful endoscopic closure of a lateral duodenal perforation at ERCP with fibrin glue. *Gastrointestinal Endoscopy*.

[11] Raju G. S., Gajula L. (2004). Endoclips for GI endoscopy. *Gastrointestinal Endoscopy*.

[12] Paspatis G. A., Dumonceau J.-M., Barthet M. (2014). Diagnosis and management of iatrogenic endoscopic perforations: European Society of Gastrointestinal Endoscopy (ESGE) position statement. *Endoscopy*.

[13] Park W. Y., Bum Cho K., Soo Kim E., Sik Park K. (2012). A case of ampullary perforation treated with a temporally covered metal stent. *Clinical Endoscopy*.

[14] Canena J., Liberato M., Horta D., Romão C., Coutinho A. (2013). Short-term stenting using fully covered self-expandable metal stents for treatment of refractory biliary leaks, postsphincterotomy bleeding, and perforations. *Surgical Endoscopy*.

[15] Odemis B., Oztas E., Kuzu U. B. (2016). Can a fully covered self-expandable metallic stent be used temporarily for the management of duodenal retroperitoneal perforation during ERCP as a part of conservative therapy?. *Surgical Laparoscopy Endoscopy & Percutaneous Techniques*.

[16] Furukawa A., Sakoda M., Yamasaki M. (2005). Gastrointestinal tract perforation: CT diagnosis of presence, site, and cause. *Abdominal Imaging*.

[17] Sung H. K., Sang S. S., Yong Y. J., Suk H. H., Jin W. K., Heoung K. K. (2009). Gastrointestinal tract perforation: MDCT findings according to the perforation sites. *Korean Journal of Radiology*.

[18] Light R. W. (1985). Exudative pleural effusions secondary to gastrointestinal diseases. *Clinics in Chest Medicine*.

[19] Croce E., Golia M., Russo R., Azzola M., Olmi S., De Murtas G. (1999). Duodenal perforations after laparoscopic cholecystectomy. *Surgical Endoscopy*.

[20] Artifon E. L. A., Minata M. K., Cunha M. A. B. (2015). Surgical or endoscopic management for post-ERCP large transmural duodenal perforations: a randomized prospective trial. *Revista de gastroenterologia del Peru : organo oficial de la Sociedad de Gastroenterologia del Peru*.

